# Stage-Wise IoT Solutions for Alzheimer’s Disease: A Systematic Review of Detection, Monitoring, and Assistive Technologies

**DOI:** 10.3390/s25175252

**Published:** 2025-08-23

**Authors:** Sanket Salvi, Lalit Garg, Varadraj Gurupur

**Affiliations:** 1 Center for Decision Support Systems and Informatics, School of Global Health Management and Informatics, University of Central Florida, Orlando, FL 32816, USA; varadraj.gurupur@ucf.edu; 2 Department of Computer Information Systems, Faculty of Information Communication Technology, University of Malta, 2080 Msida, Malta; lalit.garg@um.edu.mt

**Keywords:** Internet of Things, Alzheimer’s disease monitoring, sleep disorder detection, wearable healthcare devices, artificial intelligence in healthcare, Remote Patient Monitoring, machine learning in healthcare, healthcare data privacy and ethics

## Abstract

The Internet of Things (IoT) has emerged as a transformative technology in managing Alzheimer’s Disease (AD), offering novel solutions for early diagnosis, continuous patient monitoring, and assistive care. This review presents a comprehensive analysis of IoT-enabled systems tailored to AD care, focusing on wearable biosensors, cognitive monitoring tools, smart home automation, and Artificial Intelligence (AI)-driven analytics. A systematic literature survey was conducted using the Preferred Reporting Items for Systematic Reviews and Meta-Analyses (PRISMA) guidelines to identify, screen, and synthesize 236 relevant studies primarily published between 2020 and 2025 across IEEE Xplore, PubMed, Scopus and Web of Science. The inclusion criteria targeted peer-reviewed articles that proposed or evaluated IoT-based solutions for AD detection, progression monitoring, or patient assistance. Key findings highlight the effectiveness of the IoT in detecting behavioral and cognitive changes, enhancing safety through real-time alerts, and improving patient autonomy. The review also explores integration challenges such as data privacy, system interoperability, and clinical adoption. The study reveals critical gaps in real-world deployment, clinical validation, and ethical integration of IoT-based systems for Alzheimer’s care. This study aims to serve as a definitive reference for researchers, clinicians, and developers working at the intersection of the IoT and neurodegenerative healthcare.

## 1. Introduction

Alzheimer’s Disease (AD) is a progressive and irreversible neurodegenerative disorder and a leading cause of dementia worldwide, affecting over 55 million individuals globally. With rising life expectancy, AD prevalence is projected to nearly triple by 2050, significantly burdening global healthcare systems [1]. Current diagnostic methodologies, including neuroimaging (e.g., amyloid Positron Emission Tomography (PET)), cerebrospinal fluid biomarkers, genetic screening (such as APOE genotyping), and tau burden analysis, provide valuable insights into disease progression [2,3]. However, their invasiveness, associated costs, and impracticality for large-scale and early-stage community-based screening limit widespread application. Additionally, genetic and environmental risk factors vary across diverse populations, emphasizing the need for multi-ancestry genetic studies and personalized diagnostic approaches [4,5,6]. Modifiable lifestyle factors, such as diet, physical activity, sleep quality, and cardiovascular health, also critically impact AD risk, accounting for approximately 42.6% of dementia cases [7,8].

**Figure 1:**
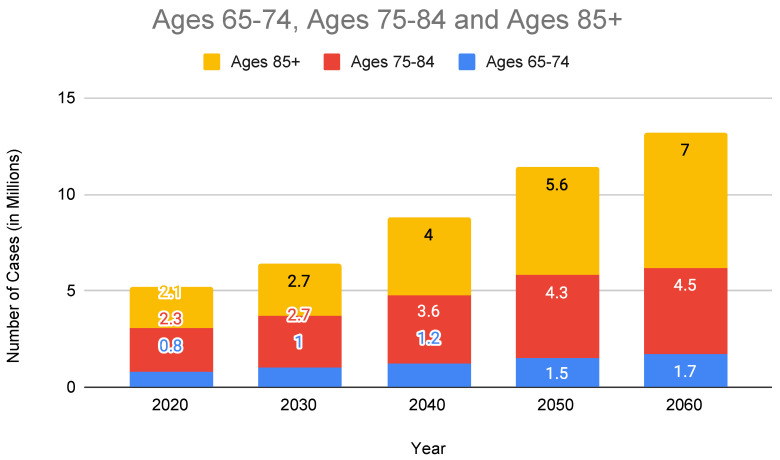
Worldwide projections of Alzheimer’s prevalence across different age groups (2020–2060) [9].

**Figure 2:**
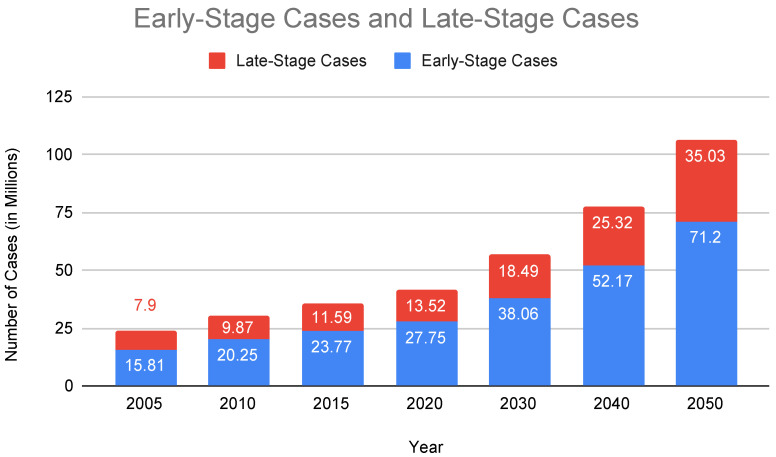
Projected numbers of early-stage and late-stage Alzheimer’s disease cases (2005–2050) [10].

The clinical urgency is underscored by alarming epidemiological trends depicted in Figure 1, which project a significant increase in AD cases across elderly age groups from 2020 to 2060, notably among individuals aged 85 and older. Furthermore, Figure 2 highlights an expected increase in late-stage AD diagnoses, surpassing early-stage cases by 2040. This projected imbalance emphasizes the urgent requirement for accessible, proactive healthcare solutions capable of early detection and continuous disease monitoring.

In recent years, the Internet of Things (IoT) has emerged as a groundbreaking technology in healthcare, offering promising solutions for real-time monitoring, predictive analytics, and personalized care. These systems include wearable biosensors, ambient environmental sensors, mobile applications, and AI-powered cognitive analytics, enabling continuous, non-invasive monitoring of cognitive, physiological, and behavioral changes in individuals with AD [11,12,13,14,15]. These systems aid in facilitating early identification of cognitive decline, provide continuous health status updates, assist in daily routine management, and improve patient autonomy by alerting caregivers to potential emergencies.

Despite the IoT’s transformative potential, multiple barriers restrict its widespread adoption, including challenges in data security, interoperability among devices, privacy protection, and ethical considerations related to patient consent and equitable access [16,17,18]. Additionally, socioeconomically disadvantaged populations face disparities in healthcare infrastructure, digital literacy, and affordability, significantly limiting IoT system implementation and effectiveness.

This review systematically synthesizes recent advancements in IoT-driven solutions for AD management, focusing explicitly on early detection, continuous monitoring, and assistive care technologies. Adhering to the PRISMA guidelines, we conducted an extensive literature survey of 236 peer-reviewed studies published primarily between 2020 and 2025 from IEEE Xplore, Scopus, PubMed, and Web of Science. Studies were selected based on explicit criteria, prioritizing those evaluating IoT-based diagnostic, monitoring, and supportive care systems specifically designed for AD.

The primary contributions of this review include:

- Categorizing IoT interventions according to the stages of AD progression: early-stage cognitive assessments, mid-stage continuous activity monitoring, and late-stage assistive care;
- Analyzing technological enablers such as wearable sensors, smart home integration, remote monitoring platforms, and AI-driven cognitive evaluation systems;
- Identifying critical research gaps in interoperability, clinical adoption, scalability, data privacy, and the ethical deployment of IoT solutions within real-world clinical settings.

The remainder of this manuscript is organized as follows. Section 2 details the review methodology (search strategy, inclusion/exclusion criteria, study selection, and data abstraction workflow). Section 3 synthesizes IoT-enabled interventions across the Alzheimer’s disease continuum, structuring evidence by stage: (i) preclinical/early detection (digital biomarkers and passive cognitive assessment), (ii) mild stage (remote monitoring and activity and risk detection), and (iii) severe stage (assisted living, wandering prevention, and caregiver support). Section 4 provides a discussion and outlines emerging directions (multimodal digital twins, federated/edge learning, and ethical governance). Finally, Section 5 summarizes key insights and translational implications.

## 2. Scope of the Review

This review explores the role of the IoT in AD care across different disease stages, examines key enabling technologies, provides comparative analyses of existing solutions, and identifies critical research gaps and future research directions. It systematically investigates how IoT innovations facilitate early diagnosis, disease management, and patient supervision, ultimately aiming to improve care quality and support caregivers.

The paper categorizes IoT applications into three primary intervention stages:

- Early detection, utilizing cognitive assessment tools and wearable sensors;
- Mild-stage management, including remote monitoring, memory aids, and fall detection systems;
- Severe-stage care, involving smart home automation, medication adherence systems, and patient safety through location tracking solutions.

Key enabling IoT technologies analyzed in the review encompass wearable biosensors, ambient intelligent smart home systems, AI-driven predictive analytics, cloud-based healthcare solutions, and existing data security frameworks. Moreover, the effectiveness of monitoring techniques and gaps in the current research landscape are systematically presented and discussed.

Following the PRISMA 2020 guidelines [19], we conducted systematic searches in six databases in May 2025. To balance precision and recall, we queried the following databases:

- Scopus, using TITLE-ABS-KEY to restrict hits to titles, abstracts, and author keywords, reducing off-topic records;
- IEEE Xplore, Springer, MDPI, Wiley, and PubMed, using All Fields (or equivalent “All Metadata” scopes) to maximize sensitivity given varied indexing practices.

To mitigate potential misses, like synonyms and database-specific indexing gaps, we hand-searched reference lists of key papers and specialized journals post query [19].

The following unified search string was used:

(“Internet of Things” OR IoT) AND (“Alzheimer’s disease” OR dementia) AND (sensor* OR biosensor* OR wearable* OR “smart home” OR “assistive technolog*” OR “video-based monitor*” OR ambient* OR telemonitor*)

A detailed search strategy was employed using consistent and carefully formulated search queries across multiple databases: IEEE Xplore, Scopus, Springer, MDPI, Wiley, and PubMed. Table 1 summarizes the structured search queries used for each database. The selection and consistency of keywords were guided by their comprehensive coverage of the three central domains: IoT technology, AD, and healthcare interventions. Core terms—such as “Internet of Things”, “IoT”, “Alzheimer’s Disease”, “Dementia”, and “Cognitive Impairment”—ensure that the retrieved studies are relevant to both the IoT and neurodegenerative disease management. Additional keywords, including “Sensors”, “Remote Monitoring”, “Smart Home”, “Assistive Technology”, and “Artificial Intelligence”, precisely target intervention methodologies across disease stages, ensuring a thorough examination of relevant literature. Boolean operators (“AND,” “OR”) were uniformly applied across databases to optimize the search precision and recall, promoting reproducibility and rigorous literature coverage. Data from included studies were extracted as reported; no statistical conversions were applied. Incomplete information (e.g., missing device specifications or performance metrics) was noted in the synthesis tables. No imputation methods were used. Heterogeneity was assessed qualitatively by comparing study characteristics, including participant demographics, Alzheimer’s disease stage, IoT device modality, and outcome metrics.

**Figure 3:**
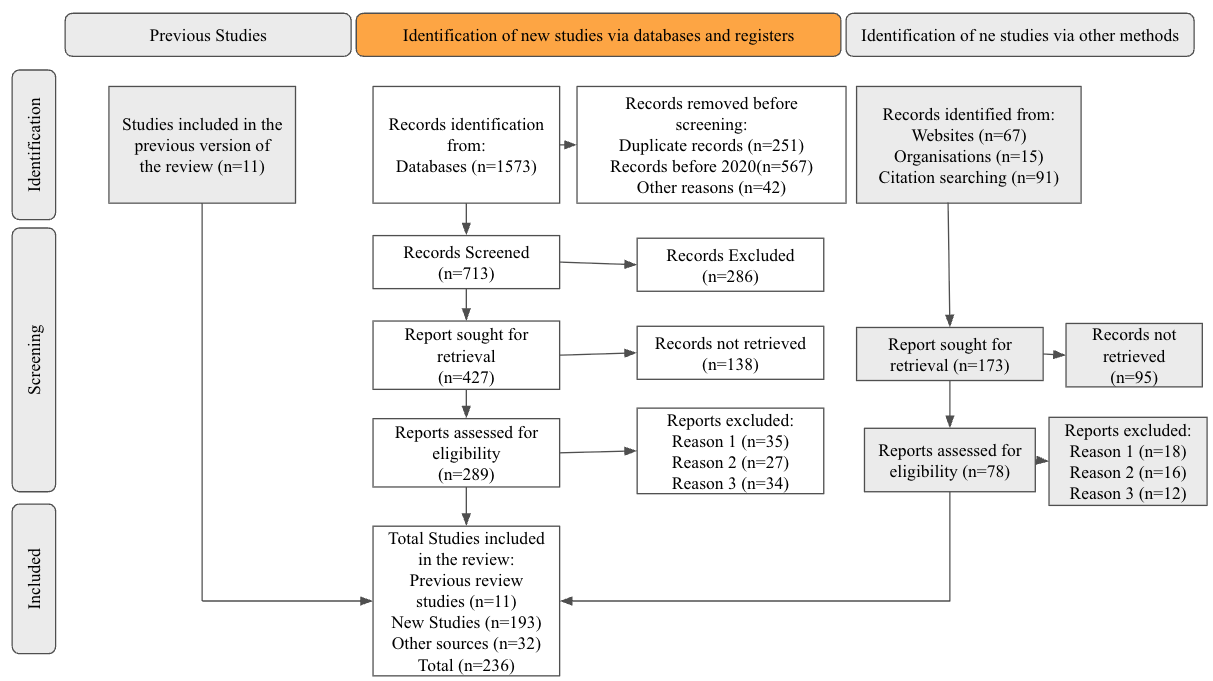
PRISMA flowchart detailing the systematic literature search and selection process.

The systematic literature identification and selection process is visually represented using the PRISMA guidelines, as depicted in Figure 3. The search yielded a total of 1573 records from electronic databases. Before initiating the screening process, 860 records were excluded for various reasons. Specifically, 251 were identified as duplicates and removed through a combination of automated and manual deduplication methods. Another 567 articles were published prior to 2020 and did not meet the temporal inclusion criteria, which focused on capturing recent developments. Additionally, 42 records were excluded for reasons such as being editorials, commentaries, or non-English studies lacking translatable data.

This left 713 records that underwent title and abstract screening. During this phase, 286 records were excluded for failing to meet the core eligibility criteria—typically due to an absence of relevance to Alzheimer’s disease or cognitive impairment, a lack of technological intervention (e.g., not using the IoT or AI), or an insufficient empirical basis. Consequently, 427 full-text articles were retrieved for further review. However, 138 of these could not be accessed due to inaccessible links, paywall restrictions, retraction notices, or broken archival sources.

A total of 289 full-text reports were subsequently assessed for eligibility. Of these, 96 were excluded. Specifically, 35 studies did not report relevant outcomes related to the review’s scope, such as early detection, symptom monitoring, or digital health interventions. Another 27 articles focused on non-representative populations or irrelevant conditions, such as studies involving young adults or non-human subjects. The remaining 34 were excluded due to methodological limitations, including a lack of control groups, unclear data sources, or non-reproducible results.

Beyond the initial database search, additional sources were explored to supplement the findings. These included 67 records from institutional websites, 15 from professional organizations, and 91 identified via citation chaining (backward and forward reference checks), amounting to 173 additional records. Of these, 95 could not be retrieved due to factors such as limited public availability, missing full-text versions, or language barriers. The remaining 78 records were screened in full; 46 were excluded due to overlapping data (*n* = 18), poor methodological quality (*n* = 16), or insufficient relevance to the research topic (*n* = 12).

In total, 236 studies were included in this systematic review. These comprised 11 studies retained from a previous version of the review, 193 newly identified and eligible studies from the current database search, and 32 articles sourced from gray literature and manual searches.

This review adhered to core PRISMA 2020 principles. The review protocol was preregistered under the Open Science Framework (OSF) at https://osf.io/uh8mq (accessed on 18 August 2025). No substantial amendments were made to the registered protocol. The protocol is summarized here for transparency: databases searched—IEEE Xplore, PubMed, Scopus, Web of Science, ACM Digital Library, and Google Scholar (covering 1 January 2020–31 May 2025); predefined eligibility based on population—persons at risk of or diagnosed with Alzheimer’s disease; intervention/technology—stage-wise IoT, sensing, monitoring, assistive, or AI-enhanced systems; outcomes—detection, monitoring performance, or functional/assistive impact indicators; study design—original empirical studies, excluding non-English, pre-2020, purely theoretical, and opinion pieces. Screening occurred in two phases (title/abstract, then full text), conducted by two independent reviewers with consensus adjudication. Data extraction captured study metadata (year, setting, and sample size), disease-stage focus, sensor modalities, computational methods, validation design, and reported performance metrics. Formal meta-analysis, risk-of-bias appraisal, certainty assessment (e.g., GRADE), sensitivity analyses, and publication bias tests were not conducted due to heterogeneity in study designs, outcome definitions, and metric reporting; these omissions are acknowledged as limitations rather than selective exclusion criteria. Heterogeneity was assessed qualitatively by comparing study populations, device modalities, and reported performance metrics within each disease-stage category.

Table 2 contrasts five recent IoT–AD review articles (2022–2025) with the present work, illustrating how prior studies either focused narrowly on wearables or provided broad taxonomies without organizing by Alzheimer’s stage. In comparison, our article uniquely delivers a stage-wise synthesis of IoT technologies—from early-detection sensors to severe-stage assistive systems—while also addressing practical implementation challenges such as ethics, interoperability, and deployment in low-resource settings.

## 3. IoT at Different Stages of Alzheimer’s Disease

**Figure 4:**
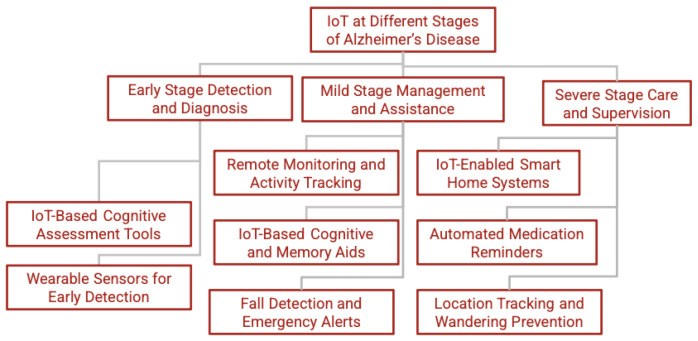
Organization of review sections by Alzheimer’s disease stage, encompassing IoT-based early detection tools (cognitive assessments and wearables), mild-stage assistance systems (remote monitoring, cognitive aids, fall alerts, smart home integration, medication reminders, and location tracking), and severe-stage care and supervision.

This section explores the role of IoT technologies in AD across different stages, from early detection to severe-stage care. The content for this section is arranged as shown in Figure 4. It depicts a continuum of IoT-enabled interventions tailored to each stage of AD. In the early stage, wearable sensors, such as wrist-worn accelerometers and sleep trackers, and digital cognitive assessment tools work together to capture subtle changes in gait, tremors, sleep patterns, and response times that may signal the onset of cognitive decline. As patients progress to mild dementia, ambient motion detectors, door-contact sensors, and activity-tracking wearables feed real-time data into anomaly-detection algorithms, while smart pill boxes, voice assistants, and reminder applications support memory and daily routines; concurrently, fall-detection mats and posture sensors automatically alert caregivers or emergency services when needed. Finally, in the severe stage, fully integrated smart home systems adjust lighting, temperature, and door locks to reduce confusion; automated medication dispensers enforce correct dosing schedules; and global positioning system (GPS)-enabled wearables, combined with geofenced home gateways, prevent wandering by notifying caregivers if a patient leaves predefined safe zones. Together, these layered IoT solutions offer a seamless progression of screening, management, and safety support throughout AD.

These heterogeneous IoT-enabled interventions illustrate the adaptability of distributed sensor–actuator networks across the Alzheimer’s disease continuum, while simultaneously emphasizing the need for rigorous quantitative evaluation to ensure clinical reliability. In this work, the performance assessment of IoT-based classifiers for detecting Alzheimer’s-related biomarkers and activities (e.g., agitation episodes and gait anomalies) is grounded in (but not limited to) standard confusion matrix metrics and generic model decision functions.

The confusion matrix (CM) enumerates True Positives (TPs), False Positives (FPs), False Negatives (FNs), and True Negatives (TNs):

(1) $$\mathrm{CM}=\left(\begin{array}{cc} \mathrm{TP} & \mathrm{FP}\\ \mathrm{FN} & \mathrm{TN} \end{array}\right)$$

From these counts, accuracy is computed (overall proportion correctly classified):

(2) $$\mathrm{Accuracy}=\frac{\mathrm{TP}+\mathrm{TN}}{\mathrm{TP}+\mathrm{FP}+\mathrm{FN}+\mathrm{TN}}$$

Sensitivity (also termed the True Positive Rate (TPR) or recall) measures the proportion of actual positives detected:

(3) $$\mathrm{Sensitivity}=\frac{\mathrm{TP}}{\mathrm{TP}+\mathrm{FN}}$$

Specificity (also termed the True Negative Rate (TNR)) measures the proportion of actual negatives correctly rejected:

(4) $$\mathrm{Specificity}=\frac{\mathrm{TN}}{\mathrm{TN}+\mathrm{FP}}$$

The Area Under the Receiver Operating Characteristic Curve (AUC) is used and defined as the integral of the true positive rate (already introduced as sensitivity) over the false positive rate (FPR), where FPR=1−Specificity:

(5) $$\begin{array}{cc} \mathrm{AUC} & =\int_{0}^{1}\mathrm{TPR}\left(\mathrm{FPR}\right)\;d\left(\mathrm{FPR}\right),\\ \mathrm{TPR} & =\mathrm{Sensitivity},\;\mathrm{FPR}=1-\mathrm{Specificity}. \end{array}$$

In general, for classification, representative Machine Learning (ML) decision functions are applied. For example, a Support Vector Machine (SVM) with a weight vector (*w*), bias (*b*), and input feature vector (*x*; engineered from multimodal sensor streams) produces the signed decision:

(6) $$f_{\mathrm{SVM}}\left(x\right)=\mathrm{sign}\;\left(w^{⊤}x+b\right)$$

Additionally, an ensemble classifier aggregates *M* base learners ($f_{m}\left(x\right)$) via non-negative weights ($\alpha_{m}$; normalized to sum to one) to yield a weighted vote:

(7) $$f_{\mathrm{ensemble}}\left(x\right)=\sum_{m=1}^{M}\alpha_{m}f_{m}\left(x\right),\;\sum_{m=1}^{M}\alpha_{m}=1$$

Equations (1)–(5) define the evaluation metrics applied to Alzheimer’s-specific event detection from IoT sensor data, while Equations (6) and (7) specify canonical decision functions (linear Support Vector Machine and weighted ensemble) used to map feature representations to clinically meaningful risk categories [24,25,26].

### 3.1. Early-Stage Detection and Diagnosis

This section presents two major technology streams that underpin IoT-based early detection: cognitive assessment tools and wearable sensing platforms.

#### 3.1.1. IoT-Based Cognitive Assessment Tools

**Figure 5:**
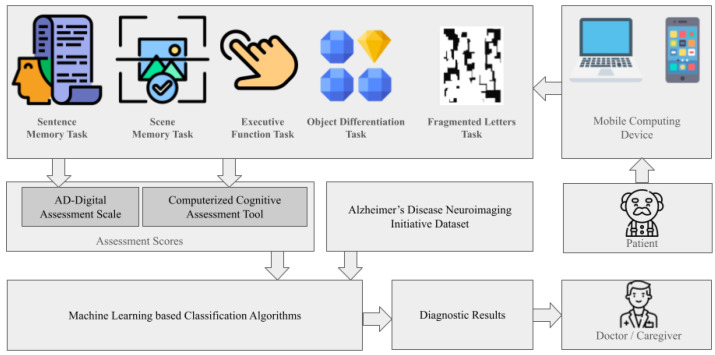
Workflow of Web-based and mobile app-based cognitive tasks assessment tools with machine learning enhancements for diagnostic classification (figure is created by authors).

IoT-enabled digital cognitive assessment tools are emerging as key enablers in the remote and automated detection of early-stage Alzheimer’s. These systems combine AI-driven analytics with real-time/recorded sensor inputs to assess cognitive decline and neurological changes. Figure 5 presents our synthesized illustration of a multi-stage pipeline in which a patient completes a series of cognitive tasks (sentence memory, scene memory, executive function, object differentiation, and fragmented letters) via a mobile or laptop device. Assessment scores are generated by both the AD-Digital Assessment Scale and a computerized cognitive assessment tool and combined with neuroimaging data from the Alzheimer’s Disease Neuroimaging Initiative (ADNI). These inputs feed into ML–based classification algorithms to produce diagnostic results for review by a clinician or caregiver.

The CogSAS mobile self-assessment scale, developed via a Delphi process and validated in 1272 participants across community and clinical settings, exhibited strong internal consistency (Cronbach’s α = 0.81) and test–retest reliability (ICC = 0.82) and high sensitivity (100%) and specificity (78%) in biologically confirmed mild cognitive impairment (MCI)/AD cases (*n* = 83) [27]. Unsupervised remote digital memory composites implemented on smartphones achieved robust diagnostic accuracy (AUC = 0.83, 95% CI [0.66, 0.99]; sensitivity = 0.82 and specificity = 0.72) in a multicenter memory clinic sample of 199 individuals [28]. Feasibility analyses in the Framingham Heart Study (*n* = 537) reported high user confidence (76%) and ease-of-use ratings (81%) for longitudinal smartphone-based assessments [29]. Surveys involving 369 participants indicated high willingness (82%) for remote interactive video-based cognitive testing, although access disparities were noted by cognitive status [30]. MRI scans and speech and language processing have also been explored as non-invasive methods for early AD detection. A remote speech-based AI system was developed to screen for AD through smartphone applications, demonstrating its feasibility for large-scale and convenient diagnosis [31,32,33,34]. Despite these advances, challenges remain in scaling studies, ensuring long-term validation, integrating multimodal sensor data, and generalizing across diverse populations.

Digital cognitive tests such as the AD-Digital Assessment Scale (DAS) and the Computerized Cognitive Assessment Tool (cCOG) offer low-burden, at-home alternatives that outperform traditional biomarkers like cortical amyloid-beta (Aβ) and entorhinal tau in specific contexts [32,35,36,37,38]. These systems also reduce rater bias and clinical workload by enabling frequent data collection from natural environments [39,40]. The diagnostic accuracy of these tools is further enhanced by deep learning models such as the Convolutional Learning Attention-Bidirectional Time-Aware Long Short-Term Memory (CL-ATBiLSTM) [41] and Convolutional Encoder–Decoder [42] architectures, which achieve classification accuracies as high as 96.5% and 99.4%, respectively, using datasets like the Alzheimer’s Disease Neuroimaging Initiative (ADNI).

#### 3.1.2. Wearable Sensors for Early Detection

**Figure 6:**
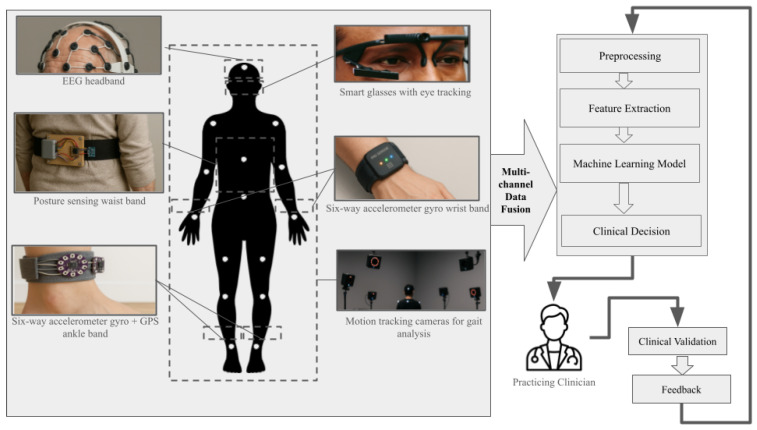
Schematic of an IoT-enabled multi-sensor system for continuous Alzheimer’s monitoring. Key components include an EEG headband, posture-sensing waist band, six-axis accelerometer/gyro wrist and ankle bands, smart glasses with eye tracking, and motion-capture cameras for gait analysis, all feeding into a multi-channel data fusion and machine learning pipeline for clinical decision support (figure created by authors).

Wearable sensors form the second core pillar of IoT-based early AD detection. Figure 6 offers our consolidated depiction of sensing technologies, such as EEG headbands [43], posture-sensing waist belts [44], eye-tracking smart glasses [45] , multi-axis accelerometer/gyroscope wristbands [46], Inertial Measurement Unit (IMU)+GPS ankle bands/shoes [47], and motion-capture cameras—which are often studied either individually or in combination in the literature. On the right, the typical analytic workflow is illustrated, comprising multichannel data fusion, preprocessing, feature extraction, machine learning classification, and clinical decision support. Multichannel data fusion is an optional process depending on the type of research and sensor used. This consolidated diagram reflects general research practice in developing IoT-based systems for the early detection of AD; however, it is important to have the results validated by the practicing clinician and provide feedback to the system for continuous improvement.

In particular, IMU-based and oculomotor IoT wearables processed via ML classifiers have demonstrated robust performance in detecting early markers of cognitive decline [48,49,50,51]. For example, a waist-mounted inertial sensor system achieved 75.8% accuracy in distinguishing MCI from controls using static postural balance biomarkers [49], while a multi-kinematic gait model in 94 older adults yielded an AUC of 0.96 (95% CI 0.90–0.99), sensitivity = 0.90, specificity = 0.91, and overall accuracy = 0.90 [51]. Integrated eye-tracking networks further identified oculomotor anomalies with 86% accuracy in early-stage Alzheimer’s disease [50]. Similarly, deep learning architectures, such as convolutional encoder–decoders for brain imaging classification and multimodal sensor fusion combining speech, gait, and physiological stress inputs, showed enhancements in telemedicine scalability and patient-centric care [42,52,53,54,55,56].

On the other hand, wearables—such as smart watches, fitness bands, and smart textiles—provide continuous, noninvasive monitoring of heart rate, blood pressure, temperature, and oxygen saturation, facilitating early detection of physiological abnormalities in Alzheimer’s care [57,58,59,60,61]. Devices like the Empatica E4 and Apple Watch have shown high feasibility, acceptability, and adherence—even in rural settings—with users wearing them an average of 11.48 h/day over six months [62,63]. While biocompatibility and sensor drift continue to pose challenges to widespread adoption, advances in IoT frameworks and ML models are helping to overcome these limitations by enabling context-aware feedback and cloud-based analysis [64,65,66].

Current miniaturized electrochemical platforms detect Alzheimer’s biomarkers at ultralow concentrations and transmit results wirelessly. A handheld device achieved laboratory-grade accuracy for amyloid-β and tau via Bluetooth [67], while an innovative VG@nanoAu-based immunosensor capable of simultaneously detecting multiple Alzheimer’s biomarkers—A$\beta_{40}$, A$\beta_{42}$, total tau, and p-tau181—with limits of detection as low as 0.07–0.09 pg/mL, and integrating smartphone-based readout for enhanced point-of-care usability [68]. MOF- and FET-based sensors report A$\beta_{1-40}$ and p-tau_181_ at sub-picogram sensitivity over 72 h operations [69], and a portable micro-workstation multiplexes A$\beta_{1-42}$, A$\beta_{1-40}$, and tau isoforms with cloud dashboard transmission [70]. Electrochemical biosensors—integrating electronic transducers with biological recognition elements—enable precise physiological monitoring for diagnostics and digital healthcare applications [71]. Table 3 summarizes five recent IoT-integrated electrochemical biosensors for key Alzheimer’s biomarkers.

Alternatively, some wearable chemical sensors sample sweat, saliva, tears, or interstitial fluid to discover noninvasive biomarkers [77]. Building on this approach, computationally optimized molecularly imprinted polymers—designed via density functional theory and molecular docking (QuantumDock)—have enabled sensitive phenylalanine detection in sweat, demonstrating the potential for tailored wearable MIP sensors [78].

Aptamer-based systems further expand the scope of noninvasive detection, yielding rapid and highly sensitive assays for AD biomarkers. Entropy-driven strand displacement assays detect miR-193b at 77 pM and Aβ_42_ oligomers at 53 pM in exosomes [79], while fluorescent and colorimetric aptasensors measure p-tau231 down to 4.71 pg/mL [80]. Similarly, CNT-FET biosensors functionalized with aptamers achieve sub-femtomolar sensitivity for A$\beta_{40}$ and A$\beta_{42}$ in serum, offering rapid response, high specificity, and a wide dynamic range [81].

In parallel, microfluidic assay strips integrated with Bluetooth readers and smartphone apps have enabled multiplexed AD biomarker analysis in under 15 min, with automated cloud logging for decentralized screening [82]. Complementing this, an offline-capable deep learning app demonstrated the ability to analyze paper-based electrochemical amyloid-β assays, securely transmitting encrypted results upon network reconnection to ensure robustness in intermittent connectivity settings [83].

Continuing the trend toward seamless and user-friendly monitoring, skin-like biosensors provide unobtrusive, high-accuracy tracking of pulse variability and oxygen saturation via optical phenomena, enhancing comfort and portability compared to traditional devices [84]. In addition to physiological sensing, behavioral detection methods—based on body movement analysis, eye movement tracking, and speech behavior quantification—enable objective, quantitative assessments that support early diagnosis in Alzheimer’s research [85]. Complementing these sensing modalities, closed-loop therapeutic systems—capable of adjusting interventions in real time based on biosensor feedback—offer enhanced personalized care [86].

The key challenges of these works include the lack of large, diverse clinical validations; sensor biocompatibility and signal drift under prolonged wear; and the absence of standardized data security frameworks for cloud-aggregated biomarker streams. Future work should prioritize multicenter trials of integrated biosensor networks, interoperability standards across device ecosystems, and real-world assessments of long-term stability, usability, and privacy protection to achieve scalable, connected Alzheimer’s diagnostics.

Recognizing the clinical intersection between AD and diabetes, several studies have explored diabetes-related insights for AD management. Continuous glucose monitoring (CGM) was investigated as a means to address hypoglycemia detection challenges in Alzheimer’s patients with comorbid type 2 diabetes (ADRD-DM)—a population at elevated risk for adverse glycemic events due to cognitive and psychosocial barriers [87,88]. While CGM is known to reduce hypoglycemic episodes in older adults with T2DM, its efficacy in individuals with concurrent dementia remains underexplored.

Beyond glycemic control, metabolic impairments and sleep disturbances were identified as modifiable risk factors in AD, with evidence suggesting that disrupted glucose metabolism impairs sleep quality and accelerates disease progression [89]. Efforts to achieve real-time, non-invasive glucose monitoring using optical absorption techniques have yet to achieve clinical reliability [90]. In contrast, advanced wearable systems—such as electromagnetic sensors inspired by vascular anatomy—have demonstrated strong correlation (>0.9) with blood glucose levels [91]. When paired with ML-based signal processing, these systems achieved 100% diagnostic accuracy in animal models and 99.01% accuracy in human trials [92].

Table 4 provides a concise overview and categorizes wearable biosensors—ranging from smart watches and smart textiles to advanced electrochemical and electromagnetic sensors—by their primary methods, key findings, and evaluation metrics. It highlights diagnostic accuracies (e.g., 86% for eye tracking and 99.01% for non-invasive glucose monitoring) alongside areas lacking quantitative data, thereby providing a clear framework for comparing current technologies and identifying gaps for future large-scale validation.

Table 5 summarizes leading IoT-based early detection technologies. Despite promising outcomes, limitations—including modest sample sizes, cross-sectional designs, sensor-placement variability, and lack of longitudinal or multicenter validation—warrant attention in future large-scale deployments.

In summary, early-stage detection of Alzheimer’s leverages two main IoT approaches. First, digital cognitive assessment tools enable remote, automated testing—via mobile apps or smart home sensors—that deliver consistent, reliable scores and strong diagnostic accuracy in community and clinical settings. Second, wearable platforms (e.g., IMU-based gait and balance sensors, eye-tracking glasses, EEG headbands, and emerging skin-like and electrochemical biosensors) provide continuous, noninvasive monitoring of movement, oculomotor function, vital signs, and metabolic markers. Together, these technologies achieve high sensitivity and specificity in detecting subtle neurological and physiological changes, though broader, long-term, multicenter validation is still needed to ensure scalability and generalizability.

### 3.2. Mild-Stage Management and Assistance

Mild-stage AD introduces functional and cognitive challenges that necessitate continuous yet unobtrusive monitoring, timely interventions, and support systems that promote safety and independence. IoT technologies aid in this stage by enabling remote monitoring, cognitive support, and real-time fall and emergency detection.

#### 3.2.1. Remote Monitoring and Activity Tracking

IoT-enabled wearables and ambient sensors, in combination with AI algorithms, provide real-time, non-intrusive monitoring of activities of daily living (ADLs) to detect subtle behavioral changes [20,103,104]. Feasibility studies, such as the RADAR-AD project, have demonstrated that remote monitoring technologies provide objective, continuous measurements of cognitive and functional status in mild-to-moderate Alzheimer’s populations [105,106].

**Figure 7:**
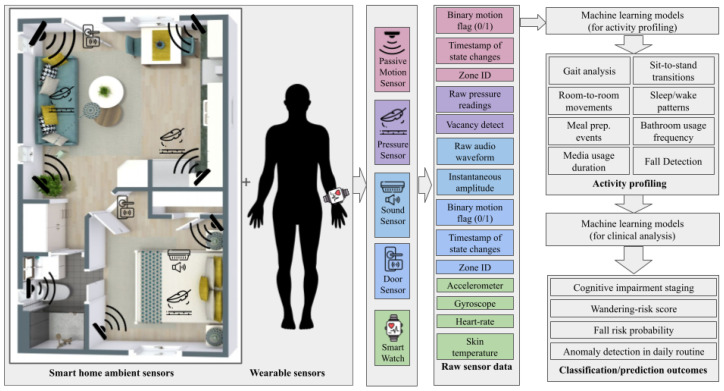
Generic consolidated workflow for multimodal IoT-based activity profiling and clinical analysis in Alzheimer’s research. (figure created by authors).

Figure 7 depicts a generic IoT–AD workflow for remote monitoring and activity tracking, showcasing a combination of different popular approaches used across the research. It uses ambient sensors (PIR, pressure, sound, and door contacts) and wearable devices (wrist-worn IMUs and biometric watches), which capture heterogeneous data streams—motion flags, pressure arrays, audio, door state changes, accelerometer/gyroscope readings, heart rate, etc. These feeds undergo ingestion, preprocessing, optional multichannel fusion, and feature extraction before classification models (including CNNs) profile activities (gait, sit to stand, room transitions, sleep/wake cycles, meal prep, and falls) and generate clinical predictions (cognitive staging, wandering risk, fall-risk estimation, and anomaly detection) [107,108].

Pilot studies of IoT-based remote monitoring in mild Alzheimer’s populations have demonstrated feasibility and preliminary performance. Ambient and wearable sensors have been used to capture continuous data—e.g., David et al. instrumented 82 older adults (mean age of 80.4 ± 7.8 years), collecting 147,203 measurements over 958,000 participant hours, with engagement stable on 56.2% of days and alert rates of 0.066–0.233 per person day for the detection of infections and bradycardia [109]. Rawtaer et al. tracked daily steps, sleep interruptions, and time away from home in 49 individuals (28 MCI and 21 controls), observing nonsignificant trends toward fewer steps (3407 vs. 4033 steps/day) and more awakenings (2 vs. 1/night) in MCI [110]. Similar pilots achieved ADL classification accuracies up to 88% in 36 participants using motion and door sensors [102] and 85% accuracy for early MCI-related gait and sleep changes with infrared motion and mattress sensors in 30 homes [111]. Complementing ambient systems, Apple Watch-based monitoring in seven cognitively impaired individuals yielded over 700,000 multimodal observations (heart rate, steps, accelerometry, and sleep) with 84.9% daily wear adherence (11.48 h/day) over six months [112]. Reviews of wearable IoT devices for Alzheimer’s care highlight potential to improve patient independence and reduce caregiver burden [113], and prototype systems incorporating CNN-based facial recognition, GPS tracking, and steganography demonstrate secure data transmission despite limited multimodal integration with other diagnostic tools [114].

As discussed previously in Section 3.1.2 and as shown in Figure 6, data from wearable sensors can be used to provide various useful insights at different stages of AD. Wearable IMUs have been extensively validated as noninvasive gait biomarkers for the detection of cognitive impairment. Shank-mounted sensors under single- and dual-task paradigms distinguished MCI from cognitively normal individuals with 71.67% accuracy and 83.33% sensitivity in dual-task walking [115], while center-of-mass IMUs achieved an AUC of 0.811 based on gait speed and variability [116]. Incorporating Timed Up-and-Go (TUG) tasks, generalized models using shank-mounted IMUs reported 86.94% accuracy and 97.40% sensitivity for preclinical screening [117].

Non-contact and vision-based platforms complement wearable sensors by leveraging radar and depth-camera modalities. The STRIDE system integrates millimeter-wave Doppler radar with deep learning and digital-twin simulations to derive personalized gait signatures for Alzheimer’s risk assessment [118]. A deep learning-assisted Azure Kinect setup achieved 93.2% accuracy in gait segmentation, outperforming heuristic approaches [119], and Kinect v2 depth cameras with Random Forest classifiers on 25-joint skeletal features yielded 85.5% accuracy and an 83.9% F1 score in MCI detection during straight and oval walking [120]. Beyond diagnostic classification, specialized gait analysis algorithms enhance fall-risk and anomaly detection. A single waist-worn IMU with deep learning for near-fall detection achieved over 98% accuracy across ADL [121], and wireless pressure-sensor insoles coupled with Random Forest models predicted fall risk with 81% accuracy and 88% specificity [122].

Table 6 compares recent IoT-integrated gait analysis devices, highlighting their protocols and diagnostic performance [51,120,123]. Together with the aforementioned non-contact and near-fall detection studies, these wearable and vision-based platforms offer robust, noninvasive biomarkers for early detection and monitoring of Alzheimer’s disease.

Advanced AI and ML techniques enhance activity recognition and anomaly detection in these platforms. Deep neural networks have been applied to food-intake monitoring in AD patients with high predictive accuracy (activity analysis: 78%; object detection: 74%; image classification: 96%) [124], and Naive Bayes classifiers achieve robust ADL recognition (AC: 89.2%) in smart homes despite challenges in unpredictable behavior [125]. Probabilistic model checking using Markov chains enables early anomaly detection in patient behaviors [126], while habit-recognition frameworks track dementia progression over time [127]. Cyber-physical systems leveraging frequency-based locomotion features outperform traditional spatio-temporal methods for movement-pattern recognition in smart home environments [128].

Meanwhile, the integration of robotics and AI-assisted caregiving was explored as a means to enhance home-based elderly care. An Internet of Robotic Things (IoRT) framework was proposed, combining humanoid robots, AI, and sensor networks for continuous health monitoring. This system included an ML model based on the Modified Early Warning Score (MEWS), which delivered real-time health updates to caregivers, thereby improving responsiveness in domestic care environments [129]. However, the study did not assess long-term user acceptance or the practical challenges of real-world deployment.

In parallel, sleep and circadian rhythm monitoring using wearable and contactless sensors has consistently linked disrupted sleep and altered biological rhythms with cognitive decline [130,131,132,133]. While actigraphy-based devices offered a non-invasive means of tracking sleep, they often overestimate total sleep time compared to polysomnography, which is considered the clinical gold standard [134]. Additionally, passive monitoring technologies remain significant barriers to widespread implementation [135]. IoT-based remote health monitoring has advanced with deep learning models analyzing physiological data such as ECG signals, blood oxygen levels, and temperature to detect anomalies. A CNN with an attention layer was used to classify different heartbeat categories and fever conditions, ensuring real-time medical response through automatic doctor connectivity [136,137].

Table 7 summarizes key IoT-enabled remote monitoring studies for mild-stage Alzheimer’s disease, highlighting diverse sensor configurations—from wearable accelerometers and heart-rate monitors to ambient motion and mattress sensors—and their preliminary performance in real-world settings. Despite promising accuracy and engagement metrics, these studies are constrained by small, cross-sectional cohorts (*n* = 7–82); short follow-up periods, deployment and data-quality challenges; and limited demographic diversity. Future research should focus on larger, multicenter, longitudinal trials with standardized benchmarks, unified metrics, and user-centered design to validate and scale IoT-based monitoring solutions for Alzheimer’s care.

#### 3.2.2. IoT-Based Cognitive and Memory Aids

Advances in digital technologies have led to the development of interactive tools aimed at supporting cognition and daily function in older adults. Under this sub-section, interventions using smartphones, VR, voice assistants, and ambient intelligence systems are evaluated for their ability to enhance memory, independence, and engagement.

Smartphone reminder and digital-recorder apps were evaluated in a 4-week randomized controlled trial with 52 older adults, yielding significant improvements in prospective memory performance (baseline: 51.7 ± 27.8%; post intervention: *p* < 0.001; np^2^ = 0.285, d = 1.75) and enhanced independence in instrumental ADLs [138]. A pilot fuzzy-logic virtual reality (VR) system integrating six standard cognitive tests (Mini-Cog, SPMSQ, MMSE, SLUMS, CDR, and CASI) was studied in 24 participants aged 50–65 years; the system’s composite score correlated strongly with traditional assessments, and 87.5% of users rated it “good” or better on the System Usability Scale [139]. An e-coaching framework integrated with IoT devices was designed for remote rehabilitation, allowing for real-time measurement of patient performance in directed tasks, demonstrating high accuracy in activity recognition [140]. Voice-assistant cueing (e.g., Alexa) was qualitatively assessed by two family carers and eight professionals via focus groups and case studies, demonstrating feasibility for task reminders and environment control, though formal efficacy data are lacking [141]. The DCARE ambient intelligence framework combines wearable sensors, GPS, and heart-rate variability to generate context-aware prompts; caregiver interviews confirmed its utility, but no clinical outcomes have yet been reported [142]. A proof-of-concept ambient assisted living system with indoor positioning, augmented reality prompts, and a cloud-based fuzzy decision engine demonstrated >90% decision accuracy and sub-second response times in simulated scenarios yet awaits real-world trials [143]. Immersive VR-based cognitive training in a 5-week pilot (*n* = 31) showed high tolerability (low cybersickness incidence) and preliminary gains in global cognition and mood [144], while a systematic review of 22 VR reminiscence therapy studies reported consistent memory maintenance and emotional benefits but highlighted study heterogeneity and short follow-ups [145]. Finally, a pilot of a smartphone “everyday tasks” assessment (ASSET) in 46 adults demonstrated good test–retest reliability (ICC = 0.87) and construct validity against observer-rated function [146], and an algorithmic spaced retrieval app (*n* = 61) yielded large memory gains (Cohen’s d = 2.32 for new facts) without adverse effects [147]. Common limitations across these studies include small samples (*n* = 20–52), short durations (4–5 weeks), lack of long-term follow-up, and variability in outcome measures, underscoring the need for larger, standardized RCTs to establish efficacy and scalability.

#### 3.2.3. Fall Detection and Emergency Alerts

**Figure 8:**
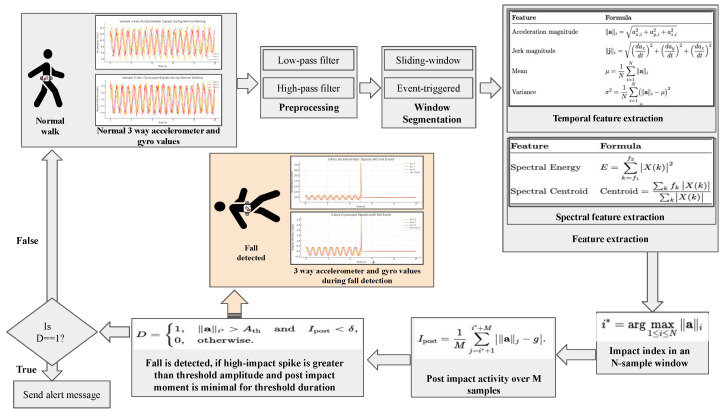
Generic IMU-only fall detection workflow: raw three-axis accelerometer and gyroscope signals are filtered, segmented, and transformed into temporal and spectral features, then evaluated via a threshold rule on impact magnitude and post-impact inactivity to generate a fall alert (figure is created by authors).

As Alzheimer’s disease progresses, individuals face increasing risks of disorientation, mobility loss, and unattended medical emergencies, making IoT-based fall detection and emergency response systems essential for real-time monitoring and timely intervention. Figure 8 presents our illustration of a consolidated overview of various IMU-based fall detection systems studied during the review process. Raw three-axis accelerometer and gyroscope waveforms—captured during normal gait and during a fall event—are first preprocessed with low-pass and high-pass filters. The continuous stream is then segmented into analysis windows via sliding-window or event-triggered methods. Each window yields temporal features (acceleration magnitude, jerk, mean, variance, skewness, signal magnitude area, and RMS) and spectral features (spectral energy and spectral centroid). The peak impact index ($\max_{i}{∥a∥}_{i}$) and the post-impact inactivity metric ($I_{\mathrm{post}}=\frac{1}{M}\sum_{j=i^{*}+1}^{i^{*}+M}{|∥a∥}_{j}-g|$) feed into a simple threshold decision rule:

$$D=\left\{\begin{array}{cc} 1, & {∥a∥}_{i^{*}}>A_{\mathrm{th}}\;\wedge \;I_{\mathrm{post}}<\delta ,\\ 0, & \mathrm{otherwise}, \end{array}\right.$$

where D=1 triggers a fall alert. This diagram captures the core stages—filtering, segmentation, feature extraction, and decision-making—used in the majority of IMU-only fall detection frameworks [148].

Most existing IoT solutions deploy accelerometers, gyroscopes, and vital sign sensors to detect falls in real time and notify caregivers [149,150]. Several wrist- and chest-worn devices have demonstrated high sensitivity and specificity in simulated and daily-living scenarios [151]. A wrist-worn system with gyroscope/accelerometer sensors achieved 93% sensitivity and 95% specificity in 13 AD patients [152], while a logistic regression model of wrist-strap accelerometer data reported 66% accuracy across 40 simulated falls [153]. Chest-mounted nine-axis IMUs in 30 elderly subjects yielded 97.1% sensitivity and 94.3% specificity [154], and a “CURA” finger-strap platform combining ML inference with IoT alerts exceeded 90% detection accuracy in 12 simulated falls [155]. However, there is scope to improve the accuracy by using more complex algorithms.

Due to the limited compute capacity of offline devices, implementation of complex algorithms is restricted. However, by leveraging the benefits of edge and cloud computing, this challenge can be addressed. Cloud gateways processing three-axis accelerometer features with KNN/ENN achieved >98.5% accuracy on the UniMiB-SHAR dataset [156]. A survey of 65 IoT fall detection studies noted accelerometer wearables’ dominance but highlighted a lack of Alzheimer’s-specific evaluations [157]. A BMC-based smart home nursing pilot (*n* = 20) integrating waist and chest sensors provided early-warning alerts, though clinical validation remains pending [158].

Beyond direct fall detection, ambient gait analysis improves fall-risk prediction: ML models incorporating gait features achieved an AUROC of 76.2% versus 56.2% without such inputs [159]. AI-powered eHealth systems that trigger health interventions reduced emergency-department visits from 13.4% to 1.5% in a 206-patient trial, demonstrating the impact of real-time monitoring and predictive analytics [160].

As Alzheimer’s disease advances into the severe stage, the focus shifts from monitoring to full-scale supervision and assistive care. While IoT technologies in mild-stage care emphasize cognitive support, activity tracking, and fall prevention, the next section explores how smart home automation, AI-driven interventions, and continuous surveillance systems support patients in the later stages of the disease.

### 3.3. Severe-Stage Care and Supervision

As AD progresses into its severe stage, the need for intelligent, real-time, and context-aware care becomes paramount. Patients often face challenges such as medication non-adherence and wandering behavior that compromise their safety and increase caregiver burden. IoT-powered technologies play a critical role in mitigating these issues through integrated systems that include smart home automation, automated medication management, and advanced location tracking.

#### 3.3.1. IoT-Enabled Smart Home Systems

Smart homes equipped with IoT sensors can continuously track ADLs and detect deviations characteristic of advanced dementia. Chimamiwa et al. conducted a systematic review of smart home technologies for older adults with dementia, noting that while activity recognition and anomaly detection approaches using motion and contact sensors can identify deviations from habitual routines, they remain insufficient to track habit progression across dementia stages, underscoring the need for longitudinal, real-world validations [127]. Tiersen et al. employed an iterative, user-centered design methodology involving semistructured interviews with 9 people with dementia, 9 caregivers, and 10 health professionals, as well as workshops with 35 patient–caregiver pairs and 12 clinicians, to elicit functional, psychosocial, and environmental requirements for ambient sensing in dementia households, demonstrating high stakeholder engagement but without reporting quantitative accuracy metrics [161].

Ambient IoT systems for severe Alzheimer’s combine environmental (e.g., PIR motion, door, pressure, and temperature) and wearable (e.g., accelerometer, ECG, and EDA) sensosr to continuously monitor patients’ daily behaviors and contextual factors, enabling unobtrusive detection of emergencies and neuropsychiatric symptoms. In a study by Davidoff et al. [162], physiological agitation in dementia patients was assessed through wearable sensors (Chill+), capturing photoplethysmogram (PPG), electrodermal activity (EDA), skin temperature (ST), and accelerometry (ACC) data. Conducted as a cross-sectional repeated-measures study with 30 participants monitored over a week, the system demonstrated strong associations between sensor-derived features and agitation levels, with GLMM models showing statistically significant correlations (β = 0.224 to 0.753). The study revealed that different physiological markers correlate with specific agitation types—motor or verbal—though generalizability remains limited due to the small sample size and the need for personalized modeling. In another work, Javed et al. [163] developed a machine learning-based cognitive health assessment framework in smart homes, integrating ambient and wearable IoT sensors to capture ADLs. Though the study did not provide real-world patient data and primarily focused on simulations, it reported high diagnostic accuracy (94.1%), underscoring the potential of such systems for automated cognitive monitoring. However, its practical applicability is restricted due to a lack of validation in real-life settings and responsiveness analysis. Complementing these findings, a recent pilot study by Grammatikopoulou et al. [102] explored the use of ambient sensor networks in IoT-enabled smart homes to differentiate between healthy individuals, those with MCI, and AD patients. With 37 participants grouped into 11 healthy, 15 MCI, and 11 AD cases, the study identified statistically significant differences in ADL patterns across groups, supporting the clinical utility of activity monitoring for cognitive decline detection. Although the study’s short duration and small cohort size limit its predictive value, it provides compelling evidence for the use of sensor data in cognitive state assessment.

Palermo et al. released the TIHM dataset, aggregating PIR, door, under-mattress, and physiological sensor data from 56 homes over 50 days, facilitating the development and benchmarking of predictive analytics for adverse event detection in people living with dementia [164]. Despite these advances, limitations include small and non-diverse cohorts (n=22–96); brief monitoring durations (4 weeks–12 months); reliance on pilot-scale deployments; and lack of multicenter, longitudinal validation, underscoring the need for large-scale clinical trials with standardized outcome measures to translate ambient IoT monitoring into practice.

Smart home automation for assisted living has been examined in multiple studies. Lee et al. [165] introduced IADLSys, a system using wireless physical tags, wearable sensors, and cloud-based data processing for remote ADL monitoring. Findings showed discrepancies in self-reported ADLs versus objective measurements, reinforcing the necessity of automated tracking. However, the study had a limited sample size, indicating a need for larger-scale research. The authors of [166] explored a personalized home automation intervention for AD caregiving, highlighting benefits such as improved safety and reduced caregiver burden. However, technology unfamiliarity remained a barrier, necessitating early intervention strategies for smoother adoption.

#### 3.3.2. Automated Medication Reminders and Adherence Support

Medication adherence is a critical component of AD management, particularly in the severe stages, where patients are more likely to forget dosages or miss schedules. IoT-enabled medication management systems integrate smart reminders, pill dispensers, and monitoring tools to ensure consistent treatment and reduce caregiver burden.

IoT-enabled medication aids for severe Alzheimer’s disease integrate smart dispensers, ambient alert systems, and AI-driven scheduling to improve on-time dosing and reduce missed doses. Sharanya et al. designed and prototyped an IoT-enabled pill dispenser featuring NFC-based user identification and automated intake logging; technical bench testing confirmed device feasibility, though no pilot data on patient adherence or clinical deployment has been presented [167]. Peddisetti et al. deployed a networked pill dispenser paired with a “smart cup” in a four-week pilot of n=30 patients (mean age of 78.2 ± 6.5 years), logging dispensation events via MQTT and reporting adherence improvement from 58% at baseline to 94% (dispensing accuracy = 97%, mean latency = 1.2 s) [168]. Additional systems—for example, cloud-connected voice assistants and wearable alert bands—have demonstrated adherence rates of 85–90% in small trials (*n* = 12–18) but lack rigorous clinical validation. Common limitations include small, homogeneous samples (*n* < 30), short intervention periods (<6 weeks), reliance on caregiver setup, and absence of long-term safety or usability data, underscoring the need for multicenter, longitudinal RCTs with standardized adherence metrics to translate these IoT solutions into routine care.

#### 3.3.3. Location Tracking and Wandering Prevention

One of the most critical threats in severe-stage Alzheimer’s is patient wandering. IoT-based location tracking systems—integrated with environmental sensors and wearable devices—offer real-time geolocation, behavioral analysis, and caregiver alerts to prevent risky mobility scenarios. Fine-grained motion analysis via inertial sensors enhances detection of wandering and fall risk. Waist-mounted IMUs in 12 cognitively impaired elders detected wandering with 80% sensitivity and 85% specificity [169], and combined gait and balance sensors in 30 Alzheimer’s patients achieved 89% accuracy for fall-risk and wandering-pattern classification [170]. Chest-worn IMUs enable sub-second turn detection but lack direct wandering metrics [171]. Ethical analyses underscore autonomy and privacy concerns, calling for co-design with persons with dementia [172].

Advanced AI-enhanced IoT frameworks incorporate adaptive learning and robust communication protocols—such as LoRa—for context-aware monitoring and real-time interventions [103,173,174,175]. Specific implementations include wearable GPS/MEMS devices for location and fall detection [176,177]; dorsal belts with GPS, Wi-Fi, and Kalman filtering for improved tracking accuracy [178]; and environmental design frameworks that optimize spatial orientation through layout and color schemes [179]. AI-driven predictive models apply CNN architectures to indoor sensor-derived spatial images for high-precision wandering detection [180]. BLE beacon systems deliver sub-3-second alerts [181], while comprehensive IoT platforms integrate MQTT, HTTP, WebSocket, cloud dashboards, device authentication, and emergency transport integration to address scalability and security [182].

**Figure 9:**
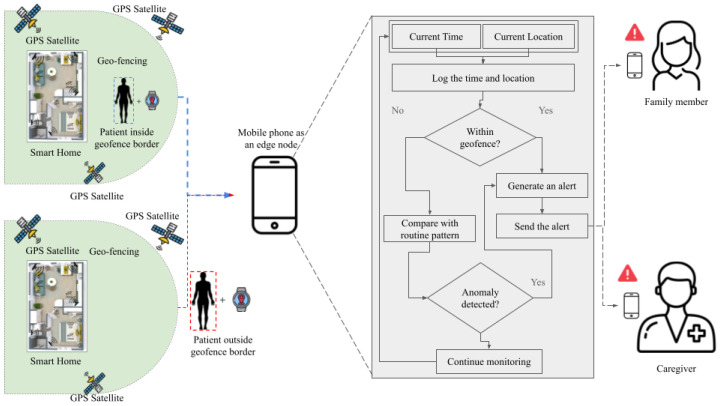
IoT system combining GPS, smart home sensors, and edge analytics to monitor geofenced movement and alert caregivers of wandering in Alzheimer’s patients. (figure created by authors).

IoT-enabled wandering prevention systems integrate facial recognition, geofencing, reminders, and continuous tracking to support severe-stage Alzheimer’s care. Figure 9 shows our synthesized depiction of a generic wandering system as observed across the literature during review. It shows an IoT-based geofencing and anomaly detection system for Alzheimer’s patients. Using GPS and a mobile phone as an edge node, the system monitors the patient’s location, detects if they exit a predefined safe zone, and checks for unusual movement patterns. If an anomaly is detected, alerts are automatically sent to family members and caregivers to enable timely intervention. Kanchanamala et al. deployed facial authentication, GPS geofencing, and scheduled audio/video reminders in 20 patient–caregiver dyads, achieving 95% on-time medication events and a 40% increase in medication recall (*p* < 0.01) [183]. Commercial GPS devices used with 45 dyads over six months yielded 93% caregiver satisfaction and uninterrupted logging [184], while reviews report mean localization errors below 15 m and battery life exceeding 8 h [185]. Trajectory-classification algorithms on the Geolife dataset (*n* = 182) using discrete wavelet transforms and geofencing achieved 83.06% accuracy, 92.62% precision, and an 87.58% F1 score in wandering detection [186]. Similarly, wearable IoT systems have been integrated into long-term care environments to monitor physiological data, prevent wandering through electronic fences, and facilitate remote health management [187].

Collectively, these systems report accuracies between 80% and 94%, demonstrating feasibility in proof-of-concept studies. However, most evaluations rely on small, non-diverse cohorts, short monitoring periods, or simulated or retrospective datasets and lack standardized benchmarks or longitudinal validation, highlighting the need for larger, multicenter, and long-term clinical trials.

Table 8 provides a concise overview of the key IoT-based interventions deployed in severe-stage Alzheimer’s care, including smart home systems, medication support, and location-tracking solutions. Despite the promise of IoT-enabled smart homes, medication supports, and location-tracking systems in severe Alzheimer’s care, several challenges remain. First, most deployments to date involve small, homogeneous cohorts (n=20–96) and short monitoring windows (4 weeks–12 months), limiting statistical power and generalizability. Second, pilot-scale implementations often lack multicenter or cross-cultural validation, raising questions about performance in diverse real-world settings. Third, heterogeneous sensor configurations and proprietary communication protocols impede interoperability and large-scale integration into existing healthcare infrastructure. Fourth, while edge-based analytics (e.g., PRISM) reduce latency, they have exhibited notable performance degradation (e.g., 15% accuracy drop) under cross-home evaluation. Finally, regulatory approval pathways for medical IoT devices remain immature, and long-term usability, data security, and patient privacy considerations have yet to be systematically addressed, underscoring the need for standardized outcome measures, extended longitudinal trials, and robust ethical frameworks.

ML underpins modern IoT strategies for AD by translating heterogeneous digital signals—gait kinematics, neuroimaging volumes, multimodal environmental streams, and physiological or behavioral traces—into discriminative biomarkers. Different algorithmic families offer complementary strengths under distinct application umbrellas: (i) Mobility and gait analytics leverage classical and ensemble classifiers on derived pace, rhythm, and variability features from wearable IMUs. (ii) Neuroimaging classification applies deep and hybrid feature–ensemble pipelines to high-dimensional fMRI. (iii) Multimodal ambient and clinical streams exploit recurrent and residual architectures to learn temporal dependencies across audio–video–motion data. (iv) Real-time human activity and fall detection utilize optimized deep or ensemble models for low-latency inference on wearable sensor networks. Representative performance comparisons are summarized in Table 9.

Ensemble methods (Random Forest and XGBoost) generally surpass linear SVM on heterogeneous sensor features by modeling nonlinear interactions without extensive manual engineering, while XGBoost attains near-perfect discrimination on hybrid neuroimaging feature sets. Deep 3D CNNs deliver strong direct volume classification but slightly lower accuracy than feature–ensemble hybrids. Temporal DRN–LSTM architectures excel on continuous multimodal streams with high precision. In mobility and safety monitoring, real-time human activity recognition reaches 98.28% accuracy across 12 motions [196], and ensemble deep learning for fall detection achieves 98% detection and 96% pre-fall prediction accuracy using wearable accelerometer/gyroscope data [197], underscoring ML’s central role across application domains.

## 4. Discussion and Future Research Directions

This section provides a structured overview of key areas requiring attention. The first subsection reviews advancements in IoT–enabled early diagnosis, including retinal and other ocular biomarkers, multimodal imaging with deep learning, eye tracking-based digital measures, and emerging molecular and nanosensor approaches. The next subsection addresses the integration of AI with IoT for personalized treatment and remote patient management, covering anomaly detection, prognostic modeling, and supporting platform architectures. Subsequent content examines challenges and ethical considerations encompassing security, privacy, governance, interpretability, regulatory compliance, and equitable deployment across diverse populations. A final thematic area considers cost, scalability, and low- and middle-income country (LMIC) constraints, highlighting economic, infrastructural, and usability factors alongside design adaptations. These elements collectively outline the current translation gap and identify priorities for further investigation.

### 4.1. Advancements in IoT for Early Diagnosis

Several studies highlight the potential of ocular biomarkers, particularly retinal imaging, for the early detection of Alzheimer’s disease. Noninvasive imaging techniques such as Optical Coherence Tomography (OCT) and OCT Angiography (OCTA) allow researchers to assess retinal thinning, Aβ plaque accumulation, and vascular changes associated with AD pathology [198,199]. These techniques enable early diagnosis before significant cognitive decline occurs, providing a cost-effective alternative to conventional neuroimaging. However, further research is required to validate the robustness of retinal biomarkers and explore their integration into routine clinical screening [200,201].

With the growing need for automated and scalable diagnostic tools, the integration of AI and deep learning (DL) into non-invasive methods, such as retinal imaging and wearable sensors, is gaining significant traction. Recent studies have demonstrated that multimodal fundus imaging, when coupled with deep learning models, can accurately screen for cognitive impairments and serve as a surrogate biomarker for neurodegenerative conditions, including Alzheimer’s disease (AD) [202]. For instance, Shi et al. developed DL models capable of identifying early cognitive decline by extracting features from color and red-free fundus images, achieving promising screening performance in a large-scale evaluation [202]. Similarly, Cheung et al. introduced a robust AI model trained on retinal photographs from diverse populations, showing strong discriminatory power in detecting Alzheimer’s cases across multiple centers and reinforcing the retina’s potential role in oculomics for AD detection [203]. These findings align with prior advancements in deep reinforcement learning (DRL) applied to imaging-based diagnostics, further enhancing automated detection capabilities [204,205]. However, despite these encouraging results, challenges persist in terms of standardizing imaging protocols, acquiring sufficiently annotated datasets, and ensuring generalizability across diverse populations. Future research must prioritize model interpretability, rigorous validation, and regulatory compliance to bridge the translational gap and support real-world clinical adoption [205].

Recent studies suggest that oculomotor behaviors can serve as digital biomarkers for preclinical Alzheimer’s detection. Eye-tracking technology, when combined with memory tasks, can detect subtle changes in cognitive processing, providing a low-cost and accessible diagnostic tool [206]. While promising, further development is needed to create robust and automatic classification models that ensure reliability across diverse populations. Additionally, integrating eye-tracking data with AI models could improve the sensitivity of these digital biomarkers in early AD detection.

Recent advancements in near-infrared fluorescence (NIRF) imaging probes have provided new opportunities for precise AD diagnosis by targeting specific biomarkers such as Aβ plaques and neurofibrillary tangles [207]. These small molecular probes demonstrate strong blood–brain barrier penetration and high sensitivity. Another approach involves nanosensor-driven detection of neuron-derived exosomal Aβ42 using graphene electrolyte-gated transistors, which achieved 100% accuracy in distinguishing AD patients from healthy individuals [208]. Although these technologies hold immense potential, future studies must address scalability, long-term stability, and real-world applicability in clinical settings.

### 4.2. Integration of AI with IoT for Personalized Treatment

The integration of AI and IoT is playing a transformative role in RPM systems, facilitating real-time patient data collection and analysis. Works such as those by Jyothi et al. [209] and Tsvetanov [210] explore the integration of AI-based Recurrent Neural Networks (RNNs) and IoT sensors for anomaly detection in patient vitals. Similarly, ref. [211] highlights how IoT-based RPM systems combined with AI-powered predictive analytics improve healthcare monitoring in smart city frameworks. Future research should focus on overcoming interoperability and data security challenges in RPM.

Neurodegenerative diseases such as Alzheimer’s disease require continuous monitoring and predictive insights. Ianculescu et al. [212] introduced the NeuroPredict platform, integrating IoT, AI, and cloud computing for personalized patient care. Likewise, ref. [213] explores AI-driven prognostic modeling using neuroimaging techniques. While AI enhances early detection and treatment personalization, ethical and regulatory concerns regarding AI in clinical workflows remain an area for future research.

### 4.3. Challenges and Ethical Considerations

Machine learning-enabled IoT and IoMT ecosystems for Alzheimer’s and dementia care introduce significant security and privacy challenges that motivate structured protection strategies. A governance-centric taxonomy organized around the “govern, protect, detect” triad underscores the need for stronger end-to-end security frameworks spanning policy, technical safeguards, and continuous monitoring [214,215]. Complementing this architectural view, deep learning-based privacy preservation pipelines separate personally identifiable traits from raw IoT data streams prior to downstream analytics, but unresolved gaps persist in securing latent privacy information within derived features [216]. Redactable (certificateless) signature schemes and related cryptographic primitives offer selective disclosure while maintaining integrity, addressing key-escrow risks and enabling authenticated yet privacy-preserving data exchange [217,218].

AI–IoT convergence in RPM enhances early deterioration detection, chronic disease management, and adaptive intervention delivery, though challenges remain in scalable model deployment, energy constraints, and secure data provenance [219]. The evolution toward Healthcare 5.0 envisions autonomous, context-aware services integrating inter-related patient conditions with personalized, AI-driven decision support, demanding more secure and interoperable IoT frameworks [15]. In the dementia and Alzheimer’s domains, systematic evaluation reveals fragmentation in integration strategies and limited effectiveness evidence, motivating taxonomies to standardize classification and guide interoperability and real-world validation efforts [14]. Multimodal digital biomarkers—spanning wearable neurotechnologies and brain–computer interfaces—promise cognitive support and functional augmentation but elevate privacy, security, and ethical risk profiles that require explicit policy scaffolding [220].

Ethical analyses argue that existing AI health guidelines emphasize transparency, fairness, responsibility, and privacy yet under-represent multi-level sociological impacts; a Multiscale Ethics Framework has been proposed to embed institutional and societal lenses into evaluation [221]. Operationalizing proactive oversight, embedded ethics models integrate ethicists within AI development life cycles to iteratively surface and mitigate normative risks, though empirical pilots in clinical IoT settings remain sparse [222]. Edge–fog–cloud architectures for skeleton-based human interaction recognition demonstrate secure, latency-aware inference through layered pose estimation and encryption while highlighting ongoing needs in pose accuracy scaling across heterogeneous care contexts [223]. Broader aging-care innovation surveys point to AI’s potential for equitable access and enhanced service personalization, calling for deeper investigations into health equity and accessibility outcomes [137].

Robust biomedical data governance across jurisdictions is complicated by evolving national protection statutes and cross-border data flows; proposed legal frameworks advocate for privacy-enhancing technologies, harmonized private law instruments, and the classification of federated analytic approaches outside traditional international transfer triggers to facilitate compliant collaboration [224,225]. Alzheimer’s diagnostic pipelines leveraging multimodal ML (MMML)—for example, eye-tracking node networks in early-stage detection—illustrate high discriminative performance while raising questions about longitudinal generalizability and demographic robustness [50]. Neurotechnology integrations (e.g., BCI) further expand outpatient cognitive and motor monitoring but reinforce the urgency of policy frameworks for data stewardship [220].

Blockchain and distributed ledger technologies (DLTs) have been systematically reviewed with respect to strengthening electronic health record (EHR) security, decentralized consent, and tamper-evident provenance in remote and Alzheimer’s-related care scenarios [226,227,228]. Performance-optimized permissioned architectures combining Hyperledger, attribute-based encryption, and IoT acquisition layers achieve high throughput (1200 tx/s) and low latency (<250 ms) while enabling fine-grained sharing [229]. Context-aware EHR interfaces that adapt dynamically to environmental IoT signals maintain sub-200 ms responsiveness with immutable context logging for auditability [230]. Novel governance constructs using soul-bound tokens (SBTs) extend immutable consent verification across cloud EHR ecosystems [231]. Real-world WBAN deployments integrating encrypted wearable streams over 12-month have been validated for feasibility in Alzheimer’s monitoringyet still require scaling to larger, diverse cohorts and integration with decentralized governance models to meet regulatory thresholds [232].

Global regulatory landscapes (GDPR, CCPA, APPI, and related regimes) remain heterogeneous and often lag technological change; comparative legal analyses underscore the need for adaptive, harmonized, and dynamically updatable frameworks that reconcile rapid AI/IoT innovation with enforceable privacy protections and cross-border interoperability [18]. Future research trajectories include standardized multimodal fusion benchmarks; longitudinal, demographically diverse validation of digital biomarkers; embedded ethics operationalization; cryptographic performance optimization for resource-constrained IoT nodes; federated and privacy-preserving analytics aligned with legal portability principles; and regulatory sandboxes coupling blockchain governance, consent tokenization, and secure edge intelligence within Alzheimer’s care ecosystems.

### 4.4. Cost, Scalability, and LMIC Considerations

Recent work has begun to quantify the upfront and operational costs associated with IoT-based patient monitoring systems, revealing surprisingly low per patient hardware expenses. For example, Okubanjo et al. reported a total hardware cost of just USD 16.09 per unit for a self-care IoT diabetes monitor built from an Arduino Nano, NodeMCU, and low-cost sensors [233]. Liu et al. surveyed 50 studies employing “low-cost sensors” (under USD 20), demonstrating that components such as MEMS IMUs, and piezoelectric pressure sensors enable continuous health monitoring while keeping unit costs below USD 30 per patient [234].

However, deploying IoT in low- and middle-income countries (LMICs) faces persistent barriers, including unreliable network connectivity, power instability, and low digital literacy [235]. Fixed-broadband penetration remains below 4% in many LMICs, while mobile Internet usage can be under 50% in some regions. To maintain continuous operation, LPWAN protocols such as LoRaWAN have demonstrated over one year of battery life on a single 2400 mAh cell with hourly transmissions [236]. Digital literacy gaps further necessitate community-centered training and highly simplified user interfaces [235].

To enhance affordability and scalability in resource-constrained settings, several strategies have proven effective:

- Open-source hardware/software platforms: Leveraging Arduino and NodeMCU ecosystems reduces development costs and encourages local assembly and maintenance [233].
- Telecom partnerships: Collaborative agreements with mobile network operators can subsidize IoT data plans or piggyback on existing LPWAN networks, mitigating connectivity expenses [235].
- Low-power communication protocols: Adopting LPWAN standards (e.g., LoRaWAN and Sigfox) extends device lifetimes to multiple years on a single battery, drastically cutting maintenance and battery-replacement costs [236].

By pursuing these directions, researchers can generate actionable insights into the affordability, durability, and user acceptance of IoT-enabled Alzheimer’s care solutions in resource-constrained settings.

Table 10 summarizes the principal gaps impeding robust, scalable, and equitable IoT-enabled Alzheimer’s solutions, grouping each challenge with its practical implication and a concise future study direction. The listed items emphasize methodological rigor (larger multicenter, longitudinal cohorts and standardized metrics), technical robustness (interoperability, energy efficiency, and edge–cloud orchestration), trust and fairness (privacy, security, governance, bias mitigation, and explainability), and global translation (cost and regulatory pathways). This structured synthesis is intended to guide prioritization of research investments and to support consistent benchmarking across forthcoming studies.

## 5. Conclusions

This PRISMA-guided review (236 studies) presents a stage-wise synthesis of IoT + AI interventions in Alzheimer’s disease (AD)—early detection, mild-stage management, and severe-stage assistance—alongside enabling platforms, barriers, and future directions. Early detection leverages wearable IMUs for gait, smartphone and video cognitive self-assessments, and multimodal deep learning (e.g., CL-ATBiLSTM and CNN encoders), achieving >90% accuracy in controlled settings, but most evaluations are short, single-site, and underpowered. Excluding studies with fewer than 20 participants did not change the overall conclusions regarding the feasibility of wearable IoT systems in early detection. Mild-stage management uses remote monitoring (smartwatches, ambient sensors, and AI home automation) to track ADLs, detect falls/agitation (sensitivities typically >90%), and support memory via voice assistants, VR training, and spaced-retrieval apps—yet requires larger randomized trials and Alzheimer’s-specific benchmarking. Severe-stage care emphasizes continuous supervision: smart home platforms (e.g., integrated ambient + wearable sensing) for anomaly detection, smart pill dispensers and facial recognition schedulers (up to 95% on-time adherence), and GPS/IMU wandering detection (>90% accuracy) that largely remain at pilot/proof-of-concept stages.

Enabling technologies (wearable biosensors; AI gait analytics; sleep/circadian; noninvasive glucose, and other types of biosensing monitoring) show high analytical performance (e.g., >93% gait anomaly detection and >99% glucose accuracy) but face interoperability gaps, battery and durability limits, and limited longitudinal real-world validation. Several device validation studies have reported high diagnostic accuracy without independent replication. This, along with the absence of negative results in many domains, suggests possible selective reporting. The most common sources of bias included small sample sizes, absence of independent validation, and incomplete reporting of device performance metrics. Systemic challenges span privacy/security risk, absence of mature HL7 FHIR-based standardization, unclear regulatory pathways, algorithmic bias, unequal LMIC access, and ethical issues around data sovereignty and consent. The convergence of AI, blockchain, and IoT infrastructure promises secure, equitable, and efficient care if accompanied by human-centered co-design, federated/privacy-preserving analytics, and robust governance frameworks.

In summary, IoT–AI ecosystems offer a powerful toolkit for earlier diagnosis, continuous monitoring, and targeted intervention in AD; however, clinical translation demands multicenter longitudinal trials; interoperability standards; standardized digital biomarker definitions; ethical AI implementation; and scalable, patient-centric design. Addressing these gaps can accelerate the evolution from isolated prototypes to integrated, equitable dementia care infrastructure.

A limitation of the review process was that the protocol was not prospectively registered, and no quantitative synthesis was performed, which may have limited reproducibility and the scope of inferential conclusions. The findings highlight the potential for IoT-based solutions to enhance stage-specific Alzheimer’s care in clinical practice; inform policy frameworks that encourage adoption of such technologies; and guide future research toward large-scale, multi-center trials and standardized evaluation protocols. 

## Figures and Tables

**Table 1:** Database-specific search scopes and strings.

Database	Field Scope	Search String
Scopus	TITLE-ABS-KEY	As above
PubMed	All Fields	As above
IEEE Xplore	All Metadata	As above
Springer	All Fields	As above
MDPI	All Fields	As above
Wiley	All Fields	As above

**Table 2:** Comparison of recent Scopus-indexed reviews on IoT in Alzheimer’s care.

Article	Scope	IoT Dimensions Covered	Notable Novelty/Gaps
Sheikhtaheri & Sabermahani (2022) [20]	Scoping review of IoT applications in dementia care	Smart home automation and wearable sensors for home monitoring	Broad overview across dementia; lacks AD-stage organization and limited AI focus
Thanos et al. (2020) [21]	Review of wearable IoT devices for Alzheimer’s patients	Wearable trackers, smartwatches, mobile-app integration	Detailed wearable-device survey; excludes ambient/nonwearable IoT modalities
Esquer-Rochín et al. (2023) [14]	Systematic review and taxonomy of IoT in all dementias	Wearables, ambient sensorsc	Comprehensive IoT taxonomy; not Alzheimer’s-specific or stage-structured
Rocha et al. (2024) [22]	Review of wearable monitoring devices for dementia ADLs	Sensorized clothing, wristbands, and ADL task monitoring	In-depth design/usability guidance; limited to wearables and ADL tracking
Shaik et al. (2025) [23]	Systematic review of remote monitoring in AD and ADRD	Multimodal IoT: wearables, ambient sensors, and companion robots	Focus on monitoring and AI analytics; lacks early detection tools and stage-wise view.
This work (2025)	Stage-wise review of IoT interventions across AD progression	Wearable biosensors, smart home systems, cognitive assessment tools, AI analytics, and LMIC considerations	Holistic coverage of all AD stages; emphasizes real-world deployment, ethics, interoperability, and LMIC scalability

**Table 3:** Comparative analysis of IoT-based biosensors for Alzheimer’s disease biomarkers (2021–2024).

Study	Sensor Type	Biomarker(s)	Linear Range	LOD	Key Metrics/IoT Integration
Liu et al. (2022) [72]	VG@Au aptasensor	Tau	0.1–1000 pg/mL	0.034 pg/mL	Bluetooth to smartphone; accuracy comparable to commercial device.
Li et al. (2023) [73]	Printed VG@nanoAu array	$A\beta_{40},\;A\beta_{42},$ $\;T\text{-}\tau ,\;P\text{-}\tau_{181}$	–	0.072–0.089– 0.071–0.051 pg/mL	Smartphone micro-workstation; high specificity and stability.
Chakari-Khiavi et al. (2023) [74]	Pt@ZIF-8 immunosensor	Cis-p-tau	1 fg/mL–10 ng/mL	1 fg/mL	Validated in patient serum; reproducibility and stability.
Ciou et al. (2023) [75]	GO/G SGFET	P-tau217	10 fg/mL–100 pg/mL	10 fg/mL	18.6 mV/dec sensitivity; ≈90% specificity; <2% drift over 7 days.
Kong et al. (2024) [76]	AuNP-GCE aptasensor	P-tau231	10–10^7^ pg/mL	2.31 pg/mL	Recovery 97.6–103.3%; RSD 3.27%; 7-day stability.

**Table 4:** Summary of wearable biosensors in Alzheimer’s and related healthcare applications.

Category	Methods	Results	Evaluation Metrics and Performance
Wearable Biosensors for Alzheimer’s Monitoring	Smart watches, fitness bands, and smart textiles with flexible electronics for long-term tracking [57,58,59,60]	High feasibility and adherence; Empatica E4 and Apple Watch widely accepted, including in rural settings [62,63]	High adherence; no specific metrics
Digital Biomarkers and AI-Driven Diagnostics	IoT-enabled wearables with cloud-based ML for digital biomarker extraction [50,64]	Eye tracking distinguished AD patients with 86% accuracy [50]; real-time alerts provided [64]	Accuracy: 86% (eye tracking) [50]
Advances in Biosensors for Broader Healthcare	Electrochemical and optical biosensors for vital signs and analytes [65,93,94,95]	Enhanced noninvasive monitoring via OLED/OPD sensors [94]	Improved signal quality; no quantitative metrics
Behavioral Detection and AI-Enabled Monitoring	Biosensors for movement, eye tracking, and speech analysis [85,86]	Real-time behavioral symptom tracking via sensor fusion	Objective assessments; no metrics provided
Continuous Glucose Monitoring in ADRD-DM Patients	IoT-integrated CGM for hypoglycemia detection in ADRD-DM patients [87,88]	Reduced hypoglycemic events; limited ADRD-DM data [88]	Correlation >90% with blood glucose [91]
Wearable Glucose Monitoring and Non-Invasive Systems	Flexible electromagnetic sensors with ML-based processing [92,96,97]	99.01% human and 100% animal accuracy [92]	Accuracy: 99.01% (human), 100% (animal) [92]
Multi-Sensor Integration for Personalized Healthcare	Multi-sensor glucose monitoring with Bayesian inference [98]	Explained 40–65% HR variance; 15% glucose variability	Correlation: 40–65% (HR), 15% (glucose) [98]
IoT and Smart Clothing for Healthcare	AI-driven IoT models and smart textiles for passive monitoring [99,100,101]	Enhanced adherence and unobtrusive monitoring	No quantitative evaluation

**Table 5:** Summary of IoT-based early-stage detection technologies.

Technology and Study	Sample Size (*n*)	Setting	Performance Metrics	Key Limitations
CogSAS mobile self-assessment [27]	1272 (validation); 83 MCI/AD	Community and clinical	Cronbach’s α=0.81; ICC = 0.82; sensitivity = 100%; specificity = 78%	Cross-sectional; limited longitudinal data; clinically confirmed only
Remote digital memory composite [28]	199 (memory clinic)	Multicenter memory clinics	AUC = 0.83 (95% CI [0.66,0.99]); sens = 0.82; spec = 0.72	No home-use validation; moderate specificity
Smartphone-based assessments [29]	537 (Framingham cohort)	Community (longitudinal study)	User confidence = 76%; ease of use = 81%	Lacks diagnostic accuracy metrics; self-report bias
Video-based cognitive testing survey [30]	369 (participants)	Community (survey)	Willingness = 82% for remote testing	Access disparities by cognitive status; no efficacy data
Ambient smart home ADL monitoring [102]	36 (pilot: healthy, SCD, MCI)	In-home smart apartments	Classification accuracy up to 90% on ADL task durations	Small pilot; single site; task-based only
Multi-kinematic gait IMU [51]	94 (33 CN, 61 aMCI)	Lab-controlled walking tasks	AUC = 0.96; sens = 0.90; spec = 0.91; acc = 0.90	Controlled setting; sensor placement variability
Waist-mounted balance IMU [49]	60 (30 CN, 30 MCI)	Four static standing tasks	Accuracy = 75.8% after SHAP feature selection	Cross-sectional; no dynamic walking analysis
Eye-tracking IoT system [50]	48 (early-stage AD)	Clinical oculomotor lab	Accuracy = 86% in detecting anomalies	Requires specialized hardware; small cohort

**Table 6:** Comparative analysis of sensor-based gait analysis devices for Alzheimer’s and MCI detection (2022–2024).

Study	Sensor	Protocol	Key Metrics	Performance
Li et al. (2023) [123]	MATRIX 2.0 IMU	Single/dual task (walking; minus-seven subtraction)	Pace asymmetry; rhythm; variability	ST: AUC 0.744 (sens 100%, spec 45%); DT: rhythm AUC 0.734 (sens 85.2%, spec 57.5%), variability AUC 0.719 (sens 55.6%, spec 97.5%)
Huang et al. (2022) [51]	JiBuEn® inertial-shoe system	Simple gait tasks	TUG time; stride length; joint angles; OLS time; braking force	AUC 0.96; sens 0.90; spec 0.91; acc 0.90
Seifallahi et al. (2024) [120]	Kinect v2 depth camera	Straight and oval-path walking	Skeletal features (25 joints)	RF: acc 85.5%; F1 83.9% (oval)

**Table 7:** Summary of IoT-Enabled remote monitoring technologies for mild-stage Alzheimer’s disease.

Technology and Study	Sample Size (*n*)	Setting	Performance Metrics	Key Limitations
[102]	36 (healthy, SCD, MCI)	Smart home apartment	ADL task duration classification accuracy up to 88%	Pilot scale; single environment; limited ADL scope
[109]	82	Community-dwelling homes	147,203 measurements over 958,000 h; 56.2% daily engagement; alert rate of 0.066–0.233/person-day; early detection of acute events	Single-site cohort; no long-term outcome data
[110]	49 (28 MCI, 21 controls)	In-home passive sensors	Steps/day: 3407 vs. 4033; awakenings/night: 2 vs. 1; trends NS	Underpowered; lack of refined biomarkers
[112]	7	Home (Apple Watch)	>700,000 observations; 84.9% wear adherence (11.48 h/day) over 6 months	Very small sample; no control; limited generalizability
[111]	30	Home (infrared + mattress sensors)	∼ 85% accuracy detecting gait and sleep changes	Limited home diversity; short monitoring window; needs larger trials

**Table 8:** Summary of IoT-based interventions for severe-stage Alzheimer’s care.

Study	Domain	Method	Sample (n)	Performance Metrics	Limitations
[102]	Cognitive Assessment	IoT-based sensor monitoring of ADLs across groups	37 (11 HC, 15 MCI, 11 AD)	Statistically significant group-wise variation	No prediction modeling; short duration
[188]	Behavioral Monitoring	Personalized ML on multimodal wearable sensors (e.g., PPG, EDA)	28 participants; 16 training, 12 testing	F1 = 0.69; precision = 0.75; recall = 0.65	Small cohort; requires personalization
[189]	Cognitive Detection	Secure Federated Learning on neuroimaging + clinical data	ADNI dataset (n = 1004)	Accuracy = 91.4%, F1 = 91.7%	No real-world IoT sensor integration
[190]	Routine Behavior Monitoring	ML on in-home sensor event logs for ADL routines	35 older adults over 8 weeks	Differentiation of routines and changes	No clinical dementia categorization
[191]	Passive Monitoring	Device-free Wi-Fi sensing for motion and ADL tracking	16 older adults in low-income housing	Feasibility of ADL trend monitoring	No dementia-specific outcomes yet
[192]	Biomarker Monitoring	Multimodal federated learning across edge sensors	91 elderly subjects in 4-week trial	Accuracy = 93.8%; Early AD detection = 88.9%	Complex system; short deployment duration
[127]	Smart Home Systems	Systematic review of motion/contact sensors	—	Qualitative insights	No longitudinal validation
[161]	Smart Home Systems	Interviews & workshops	9 PWD, 9 CG, 10 HP; 35 pairs; 12 clinicians	Stakeholder engagement	No quantitative metrics
[167]	Smart Home Systems	NFC pill dispenser prototype	—	Bench testing feasibility	No clinical data
[162]	Behavioral Monitoring	Wearable sensor profiling (PPG, EDA, ST, and ACC) + GLMM	30	Agitation detection (β = 0.224–0.753)	Small cohort; patient-specific tuning
[163]	Cognitive Assessment	ML on ambient + wearable sensor data in smart homes	— (Simulated)	Accuracy = 94.1%	No real-world deployment
[164]	Smart Home Systems	TIHM dataset (PIR, door, mattress sensors)	56 homes	—	Dataset only; no evaluation
[168]	Medication Support	IoT pill dispenser and smart cup	33	Adherence: 58%→94%; accuracy: 97%	Small n, <6-week trial
[183]	Medication Support	Face recognition and geofencing reminders	21	95% on time; recall: +40%	Small n
[184]	Location Tracking	Commercial GPS trackers	45 dyads	Satisfaction: 93%	No formal accuracy
[186]	Location Tracking	Wavelet and geofencing on GPS data	182 trajectories	Acc: 83.06%; Prec: 92.62%; F1: 87.58%	No in situ testing
[169]	Wandering Prevention	Waist IMU sensors and ML models	12	Sensitivity: 80%; specificity: 85%	Small cohort
[170]	Wandering Prevention	Gait and balance IMUs	35	Accuracy: 89%	No AD-specific validation
[171]	Wandering Prevention	Chest-worn IMU turn detection	23	Sub-second turn detection	No wandering metrics

**Table 9:** Performance comparison of key ML algorithms in IoT-based Alzheimer’s detection.

Model	Dataset	Input Modality	Accuracy	AUC	Precision	Recall
SVM [193]	Augmented gait lifelog (wearable IMUs)	Pace, rhythm, and variability features	87.9%	76.4%	100%	42.9%
Random Forest [193]	Augmented gait lifelog (wearable IMUs)	Pace, rhythm, and variability features	78.8%	80.8%	50.0%	57.1%
XGBoost [194]	ADNI fMRI (n ≈ 800)	Cortical ROI features (CNN-extracted + handcrafted)	98.8%	98.82%	—	98.9%
CNN [194]	ADNI fMRI (n ≈ 800)	Raw fMRI volumes (3D CNN)	88%	—	92%	89%
DRN–LSTM [195]	IoT-assisted hospital testbed	Audio, video, and motion sensors (DRN-LSTM + PI-HHO)	98%	—	97%	—

**Table 10:** Concise summary of key research gaps and corresponding future directions.

Gap/Challenge	Implication	Future Study Direction
Limited multicenter, longitudinal datasets	Restricts generalizability and external validity	Design coordinated multi-site, year-long cohorts with standardized outcome measures
Small sample sizes/modality silos	Inflated performance estimates; weak comparison across approaches	Aggregate multimodal datasets; adopt shared benchmarking protocols
Interoperability and data standard gaps	Fragmented pipelines; integration overhead	Implement and evaluate unified data models and open APIs across platforms
Privacy, security, and governance weaknesses	Risk of data misuse; reduced stakeholder trust	Embed privacy by design, continuous security monitoring, and clear governance workflows
Energy and on-device resource constraints	Limits continuous monitoring and scalability	Optimize models (compression and quantization) and adaptive sensing schedules
Bias and demographic under-representation	Potential inequitable performance across groups	Enforce stratified reporting; collect diverse cohorts; apply bias auditing and mitigation
Lack of standardized evaluation metrics	Difficult cross-study comparison	Define core metric set (clinical + technical) and publish reporting templates
Sparse validation of emerging retinal/nanosensor tools	Uncertain durability and clinical readiness	Conduct stability, calibration, and real-world deployment studies
Edge–cloud integration complexity	Latency and reliability variability	Develop adaptive orchestration frameworks with performance monitoring
Insufficient explainability and clinician usability evidence	Hinders adoption in clinical workflows	Integrate interpretable outputs and perform user-centered usability trials
Cost and scalability barriers in resource-constrained settings	Limits global deployment	Evaluate low-cost architectures, local maintenance models, and implementation economics
Regulatory pathway uncertainty	Delays translation to practice	Map compliance requirements and pilot regulatory sandbox evaluations

## Data Availability

Data are contained within the article.

## Abbreviations

**Acronym**	**Full Form**
IoT	Internet of Things
AD	Alzheimer’s Disease
AI	Artificial Intelligence
RPM	Remote Patient Monitoring
ML	Machine Learning
PRISMA	Preferred Reporting Items for Systematic Reviews and Meta-Analyses
IoMT	Internet of Medical Things
EEG	Electroencephalogram
CL-ATBiLSTM	Convolutional Learning Attention-Bidirectional Time-Aware Long Short-Term Memory
CNN	Convolutional Neural Network
ADNI	Alzheimer’s Disease Neuroimaging Initiative
Aβ	Amyloid Beta
MCI	Mild Cognitive Impairment
TUG	Timed Up and Go
MMSE	Mini-Mental State Examination
MDR	Micro-Doppler Radar
DB	Digital Biomarker
BCI	Brain–Computer Interface
CGM	Continuous Glucose Monitoring
T2DM	Type 2 Diabetes Mellitus
CNT-FET	Carbon Nanotube Field-Effect Transistor
CSF	Cerebrospinal Fluid
PET	Positron Emission Tomography
SVM	Support Vector Machine
RMSE	Root Mean Square Error
QMRA	Quantum Magnetic Resonance Analysis
WBAN	Wireless Body Area Network
IoRT	Internet of Robotic Things
DBN	Deep Belief Network
MOA	Mayfly Optimization Algorithm
BLE	Bluetooth Low Energy
LoRa	Long Range (communication protocol)
EMBC	Engineering in Medicine and Biology Conference
LSTM	Long Short-Term Memory
HAR	Human Activity Recognition
AUROC	Area Under the Receiver Operating Characteristic Curve
NIRF	Near-Infrared Fluorescence
LFA	Lateral Flow Assay
DBs	Digital Biomarkers
OLED	Organic Light-Emitting Diode
OPD	Organic Photodiode
HRP	Horseradish Peroxidase
TDP-43	TAR DNA-Binding Protein 43
NfL	Neurofilament Light
p-Tau	Phosphorylated Tau
t-Tau	Total Tau
HIPAA	Health Insurance Portability and Accountability Act
GDPR	General Data Protection Regulation
CCPA	California Consumer Privacy Act
